# Delta Opioid Receptor in Astrocytes Contributes to Neuropathic Cold Pain and Analgesic Tolerance in Female Mice

**DOI:** 10.3389/fncel.2021.745178

**Published:** 2021-09-16

**Authors:** David Reiss, Hervé Maurin, Emilie Audouard, Miriam Martínez-Navarro, Yaping Xue, Yann Herault, Rafael Maldonado, David Cabañero, Claire Gaveriaux-Ruff

**Affiliations:** ^1^Université de Strasbourg, CNRS, INSERM, Institut de Génétique et de Biologie Moléculaire et Cellulaire, Illkirch, France; ^2^Laboratory of Neuropharmacology, Department of Experimental and Health Sciences, Universitat Pompeu Fabra, Barcelona, Spain; ^3^Institute of Research, Development and Innovation in Healthcare Biotechnology of Elche (IDiBE), Universidad Miguel Hernández Elche, Alicante, Spain; ^4^Ecole Supérieure de Biotechnologie de Strasbourg, Illkirch, France

**Keywords:** delta opioid receptor, astrocyte, pain, cold allodynia, analgesia, tolerance

## Abstract

**Background**: The delta opioid receptor (DOR) contributes to pain control, and a major challenge is the identification of DOR populations that control pain, analgesia, and tolerance. Astrocytes are known as important cells in the pathophysiology of chronic pain, and many studies report an increased prevalence of pain in women. However, the implication of astrocytic DOR in neuropathic pain and analgesia, as well as the influence of sex in this receptor activity, remains unknown.

**Experimental Approach**: We developed a novel conditional knockout (cKO) mouse line wherein DOR is deleted in astrocytes (named GFAP-DOR-KO), and investigated neuropathic mechanical allodynia as well as analgesia and analgesic tolerance in mutant male and female mice. Neuropathic cold allodynia was also characterized in mice of both sexes lacking DOR either in astrocytes or constitutively.

**Results**: Neuropathic mechanical allodynia was similar in GFAP-DOR-KO and floxed DOR control mice, and the DOR agonist SNC80 produced analgesia in mutant mice of both sexes. Interestingly, analgesic tolerance developed in cKO males and was abolished in cKO females. Cold neuropathic allodynia was reduced in mice with decreased DOR in astrocytes. By contrast, cold allodynia was exacerbated in full DOR KO females.

**Conclusions**: These findings show that astrocytic DOR has a prominent role in promoting cold allodynia and analgesic tolerance in females, while overall DOR activity was protective. Altogether this suggests that endogenous- and exogenous-mediated DOR activity in astrocytes worsens neuropathic allodynia while DOR activity in other cells attenuates this form of pain. In conclusion, our results show a sex-specific implication of astrocytic DOR in neuropathic pain and analgesic tolerance. These findings open new avenues for developing tailored DOR-mediated analgesic strategies.

## Introduction

Chronic pain affects one-third of the human population worldwide and constitutes a major global burden of disease (GBD 2017 Disease and Injury Incidence and Prevalence Collaborators, 2018). It is a complex pathology that involves neurons, glial cells, and immune cells (Grace et al., 2014; Ji et al., 2019). Astrocytes are the most abundant cell type in the nervous system, have multiple activities, and are associated with brain pathologies (Sofroniew, 2020). They contribute to chronic pain (Ji et al., 2019). Opiates targeting mu opioid receptor (MOR) are the most frequently used analgesics for the control of moderate to severe pain. Activation of this receptor induces antinociception (elevation of nociceptive level in the absence of pain) as well as antihyperalgesia (reversal of pain hypersensitivity back to normal sensitivity levels). However, morphine and other opiates produce adverse effects including constipation, nausea, respiratory depression, and addiction. By contrast, activation of the delta opioid receptor (DOR) leads to antihyperalgesia and antiallodynia with absent or limited antinociceptive effect, indicating that DOR activity can be more effective for controlling chronic pain over acute pain perception. Agonists of DOR produce analgesia in chronic pain models (Berthiaume et al., 2020). The impact of endogenous DOR activity on pain has been explored by either administration of DOR antagonists or by the study of DOR mutant mice (Maldonado et al., 2018). Notably, DOR knockout (KO) mice with *Oprd1* gene inactivation in the whole body or peripheral nociceptive neurons showed increased hyperalgesia in neuropathic and inflammatory models (Nadal et al., 2006; Gaveriaux-Ruff et al., 2008, 2011; Martínez-Navarro et al., 2020). A number of DOR-targeted ligands have been developed for analgesia (Spahn and Stein, 2017; Berthiaume et al., 2020) and recently an oral DOR agonist was well tolerated in the phase I clinical trial (Fossler et al., 2020). Globally, this shows that activation of DOR by both endogenous opioid peptides and exogenous molecules leads to analgesia. However, whether DOR in astrocytes plays a role in pain control remains to be determined.

To investigate the function of DOR in astrocytes, we have developed a conditional KO (cKO) mouse line by using the GFAP-Cre mouse line as an astrocyte deleter line (Bajenaru et al., 2002; Eijkelkamp et al., 2010; Robin et al., 2018). The contribution of astrocytic DOR in neuropathic hypersensitivity was evaluated in these mice with reduced DOR in astrocytes. Both mechanical and cold allodynia were investigated, as cold allodynia is a clinical problem that is not frequently evaluated and the role of DOR in different cell populations has hardly been assessed until now. The implication of astrocytic DOR in analgesia and analgesic tolerance to the DOR agonist SNC80 was also investigated. In addition, as sex differences are an important factor for chronic pain and the role of astrocytes in this sexual dimorphism is unclear (Mogil, 2020; Gregus et al., 2021), and therefore the role of astrocytic DOR was investigated in both males and females.

## Materials and Methods

### Experimental Subjects and Ethical Approval

All experiments were carried out in strict compliance with the EU Directive 2010/63/UE. The experiments on the GFAP-DOR-KO mouse line were performed in IGBMC and were approved by the local ethical committees, Com’Eth, Comité d’Ethique pour l’Expérimentation Animale IGBMC-ICS with agreement number 00876-02. Mice were bred at the ICS (Institut Clinique de la Souris) animal facility and behavioral experiments were performed in the IGBMC animal facility. The experiments on the CMV-DOR-KO and DOR-flox lines were performed in the Laboratory of Neuropharmacology, Department of Experimental and Health Sciences, Universitat Pompeu Fabra, Barcelona, Spain and were approved by Comité Etico de Experimentacion Animal del PRBB (CEEA-PRBB) with agreement number DAM-4917 for the CMV-DOR-KO mouse line. Animals were housed under standard temperature, light, and humidity conditions (21 ± 1°C, 12 h light-dark cycle, 55 ± 10% humidity). Cage bedding was from Anibed (Pontvallain, France; reference AB3) and food from SAFE (Augy, France; reference D04). Food and autoclaved tap water were available *ad libitum*. Two to five mice were housed in each cage. They were habituated to the experimental environment for 10 days and to handling three times before starting the experiments. Particular effort was made to minimize the number of mice and the pain they experienced. Studies are presented according to the ARRIVE Guidelines for reporting experiments involving animals.

### Generation of the GFAP-Oprd1^f/f^ Mouse Line

The astrocyte-specific *Oprd1* knockout mouse line was generated using the CRE/loxP system as previously described (Gaveriaux-Ruff et al., 2011). Mice carrying the floxed *Oprd1* allele *Oprd1^fl/fl^*[Oprd1^tm1.1Cgrf^ allele MGI nomenclature (Gaveriaux-Ruff et al., 2011)] were crossed with *GFAP^Cre^* mice (Tg(GFAP-cre)8Gtm allele MGI nomenclature (Bajenaru et al., 2002) to obtain *GFAP^Cre^-Oprd1^fl/fl^* and *Oprd1^fl/fl^* littermates, named in this study GFAP-DOR-KO and DOR-flox littermates, respectively. GFAP-DOR-KO were compared to their DOR-flox littermates throughout the study. GFAP-DOR-KO showed no gross anatomical or behavioral defects. The genetic background of this line was 50% C57/BL6J: 50% 129svPas. Genotypes for the *Oprd1-flox* and *Cre* genes were determined by PCR on genomic DNA (35 cycles at 94°C for 30 s, 62°C for 30 s, and 72°C for 60 s). Primer sequences were: Oprd1-flox, Fwd—GTTACTGGAGAATCCAGGCCAAGCC, Rev- TGCTAGAACCTGCGGAGCCACA; Cre, Fwd—GTGTCCAATTTACTGACCGTACAC, Rev, CTAATCGCCATCTTCCAGCAG.

### Whole DOR-KO *CMV^Cre^-Oprd1^f/f^* Mouse Line

The *CMV^Cre^-Oprd1^f/f^* mice with whole body *Oprd1* gene inactivation were generated previously and genotyped as described previously (Gaveriaux-Ruff et al., 2011; Martínez-Navarro et al., 2020). They are named CMV-DOR-KO in this study.

### Analysis of *Oprd1*-Flox Gene Inactivation

The analysis of floxed *Oprd1* gene deletion was performed as described previously (Gaveriaux-Ruff et al., 2011) by PCR analysis of genomic DNA (35 cycles at 94°C for 30 s, 68°C for 30 s, and 72°C for 1 min). Primer sequences were: *Oprd1*, Fwd—GGTTAG CCTTCTGAGGGCTGGG, Rev—CCT GGC CAG CCA GTT CAC AAT CT. PCR products were separated on an agarose gel and product sizes were determined using the GeneRuler DNA ladder Mix and the GeneRuler Ultralow Range DNA ladder (SM0331; SM1213, Thermofisher, Illkirch, France). Sizes of Oprd1-flox and excised bands were 900 bp and 170 bp, respectively.

### Isolation of Astrocytes

In order to determine the selective gene deletion in astrocytes, we isolated astrocytes from the brain by using magnetic beads separation. Astrocytes were isolated from GFAP-DOR-KO and DOR-flox adult mice as described previously (Holt and Olsen, 2016). Briefly, mice were deeply anesthetized by intraperitoneal (ip) administration of 100/10 mg/kg ketamine/xylazine (Virbac, Carros, France; Rompun, Bayer, La Garenne Colombes, France) and were intracardially perfused with ice-cold Phosphate Buffer Saline (PBS) solution. Brains without a cerebellum and olfactory bulb were then placed in a culture dish, cut into small pieces, and digested with the Neural Tissue Dissociation Kit (P, 130-092-628, Miltenyi Biotech, Germany) for 30 min at 37°C. Tissue debris was removed through a 70-micrometer cell strainer. Myelin was removed by centrifugation in 30% Percoll PLUS (GE Healthcare) for 10 min at 700 g without brake. Cells were then incubated with ACSA-2 magnetic beads (130-097-678, Miltenyi Biotech, Germany) for 15 min at 4°C and separated with a magnetic field using an LS column (Miltenyi Biotech, Germany). The volume of magnetic beads used was calculated based on the number of cells counted after myelin removal. Both the ACSA-2-negative (effluent) and ACSA-2-positive (eluate, astrocytes) were collected and used for expression assays with RT-qPCR. We assessed astrocyte enrichment by analyzing the expression of the astrocyte marker *Gfap* and of the neuronal marker *Tubb3*.

### Quantitative RT-PCR

Brain areas or cell preparations were isolated and flash-frozen in liquid nitrogen and stored at −80°C. The numbers of males and females of each genotype from each brain area were as follows: dorsal root ganglia, DOR-flox, *n* = 4 males, *n* = 6 females; GFAP-DOR-KO, *n* = 5 males, *n* = 7 females; spinal cord, DOR-flox, *n* = 5 males, *n* = 5 females; GFAP-DOR-KO, *n* = 5 males, *n* = 8 females; olfactory bulb, DOR-flox, *n* = 4 males, *n* = 6 females; GFAP-DOR-KO, *n* = 5 males, *n* = 7 females; cortex, DOR-flox, *n* = 6 males, *n* = 9 females; GFAP-DOR-KO, *n* = 4 males, *n* = 5 females; hippocampus, DOR-flox, *n* = 6 males, *n* = 7 females; GFAP-DOR-KO, *n* = 4 males, *n* = 6 females; caudate putamen, DOR-flox, *n* = 6 males, *n* = 6 females; GFAP-DOR-KO, *n* = 4 males, *n* = 3 females; periacqueductal gray, DOR-flox, *n* = 12 males, *n* = 5 females; GFAP-DOR-KO, *n* = 9 males, *n* = 4 females; brainstem, DOR-flox, *n* = 6 males, *n* = 8 females; GFAP-DOR-KO, *n* = 7 males, *n* = 6 females. Quantitative RT-PCR was performed on tissues and isolated astrocyte RNA as described (Reiss et al., 2020). Briefly, total RNA was extracted with a Nucleospin kit (Macherey Nagel, Hoerdt, France) and precipitated overnight with acetate/ethanol. RNA concentrations were measured with a Nanodrop spectrophotometer (ND-1,000) and 1 μg of total RNA was reverse-transcribed in a final volume of 10 μl. Real time PCR was performed with the Light-Cycler-480 (Roche, Mannheim, Germany). The primers were: *Oprd1*, Fwd—AAGTACTTGGCGCTCTGGAA, Rev—GCTCGTCATGTTTGGCATC; *Tubb3*, Fwd—CGCCTTTGGACACCTATTCAG, Rev- TTCTCACACTCTTTCCGCAC; *Hprt*, Fwd—GGTCCTTTTCACCAGCAAGCT, Rev—TGACACTGGTAAAACAATGCA. Relative expression ratios (*Gfap* vs. *Tubb3; Oprd1* in GFAP-DOR-KO vs. DOR-flox samples in eluates and effluents) were calculated by using *Hprt* as the reference gene and the 2-ΔΔCt method to determine gene expression levels.

### Treatment Procedures

Behavioral tests were performed on mice of both sexes aged 7–15 weeks and blinded for genotype and treatment. Animals were allocated to experimental groups according to sex and genotype. Littermates were randomly assigned to experimental groups. The number of animals per group was designated in accordance with previous similar studies (Gaveriaux-Ruff et al., 2011; Martínez-Navarro et al., 2020; Tertil et al., 2021). The numbers of DOR-flox and GFAP-DOR-KO males and females of each genotype for each experiment were as follows: for assessment of mechanical sensitivity and neuropathy-induced allodynia, DOR-flox, *n* = 16 males, *n* = 16 females; GFAP-DOR-KO, *n* = 12 males, *n* = 11 females; for evaluation of SNC80-induced analgesia and analgesic tolerance, DOR-flox, *n* = 13 males, *n* = 10 females; GFAP-DOR-KO, *n* = 11 males, *n* = 7 females; for assessment of cold sensitivity and neuropathy-induced allodynia, DOR-flox, *n* = 14 males, *n* = 16 females; GFAP-DOR-KO, *n* = 14 males, *n* = 10 females. For CMV-DOR-Cre and DOR-flox controls, cold sensitivity, and neuropathy-induced allodynia, the numbers were: DOR-flox, *n* = 23 males, *n* = 17 females; GFAP-DOR-KO, *n* = 16 males, *n* = 10 females. Data were obtained from 4–8 cohorts/experiment. SNC80 (Cat. No. 0764, Tocris Bioscience, Bristol, UK) was dissolved in 0.9% saline solution with 2 microliters of 1 M HCl per mg SNC80 was administered intraperitoneally (ip). The control saline solution was injected ip using 100 microliters solution per 10 g body weight. For analyzing SNC80-induced analgesia and analgesic tolerance, the mice were treated once a day for 5 days, starting from 20 days following partial ligation of the left common sciatic nerve surgery (pSNL, see below), and tested for mechanical sensitivity threshold 45 min following each daily injection.

### Behavioral Studies

Behavioral studies were performed on a normal light-dark cycle and conducted between 8 a.m. and 3 p.m. in isolated, light and temperature-controlled rooms. Nociceptive basal levels were determined as previously described (Gaveriaux-Ruff et al., 2011). Briefly, sensitivity to touch and mechanical allodynia was determined by using Von Frey filaments (Bioseb, Vitrolles, France) applied under the mouse hindpaw and following the up and down method. Cold allodynia was determined with the cold plate apparatus (Bioseb, Vitrolles, France). Each mouse was placed on the plate (5°C) for a 5 min period, and the number of paw lifts was counted. For baseline response to cold, the mean responses of both paws were calculated. Neuropathic pain was induced by pSNL by using a 7-0 braid silk suture under ketamine/xylazine anesthesia (100/10 mg/kg mixture; ketamine, Virbac, Carros, France; xylazine, Rompun, Bayer Healthcare, La Garenne Colombes, France) according to the method previously described (Martínez-Navarro et al., 2020). Because it was shown that sham mice did not experience hypersensitivity (Gaveriaux-Ruff et al., 2011; Martínez-Navarro et al., 2020), and to decrease the number of experimented animals, we used pSNL animals only in this study.

### Statistics

Statistical analyses were performed with GraphPad Prism version 9 (San Diego, CA, USA). The normality of the data was tested using the D’Agostino-Pearson test. Afterward, the statistical significance of differences between means was determined using ANOVA followed by Sidak’s *post hoc* tests, mixed linear analysis, using Mann-Whitney or Wilcoxon tests when appropriate. *P*-values of 0.05 or less were considered significant. The sample size was chosen based on similar research studies in the field (Gaveriaux-Ruff et al., 2011; Martínez-Navarro et al., 2020). The number of animals used are indicated in the figure legends. Statistical analyses are shown in Supplementary Tables.

## Results

### Generation and Characterization of Mice With Astrocyte-Specific Oprd1 Deletion

**Figure 1:**
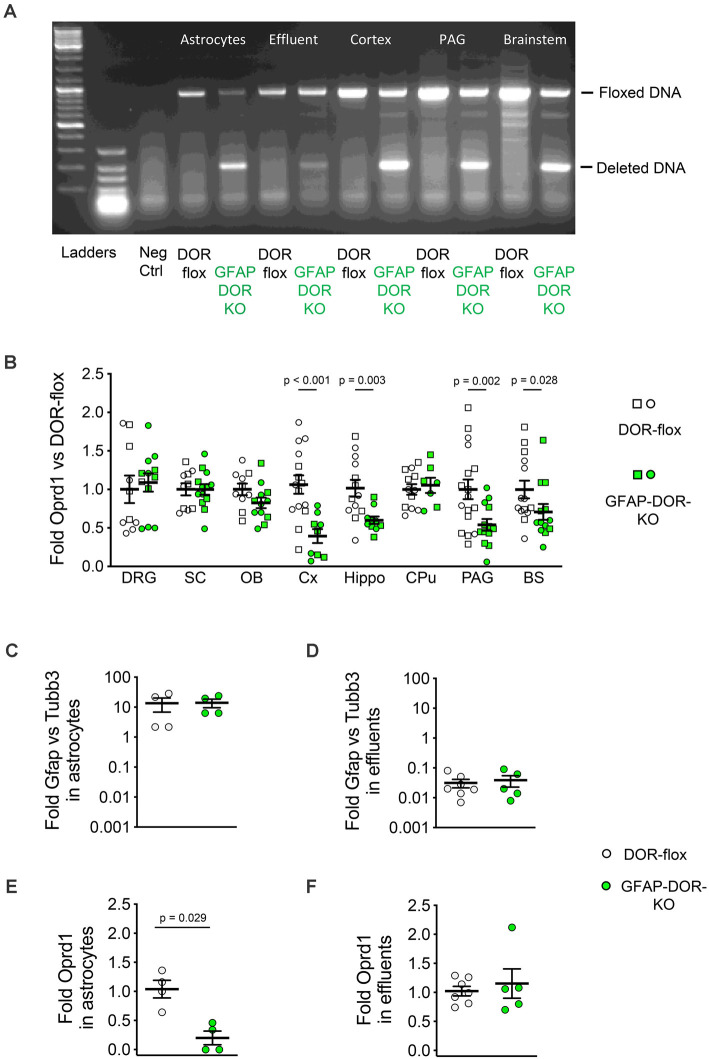
Characterization of the GFAP-DOR-KO mouse model for astrocyte-specific deletion of Oprd1. **(A)** PCR on genomic DNA shows the deletion of the floxed *Oprd1* (DOR) gene brain from GFAP-DOR-KO mice and no deletion in the brain from control DOR-flox mice. **(B)** RT-qPCR analysis shows reduced *Oprd1* expression in the cortex (Cx), hippocampus (Hippo), periacqueductal gray (PAG), and brainstem (BS) of GFAP-DOR-KO animals. DRG, Dorsal root ganglia; SC, spinal cord; OB, olfactory bulb, CPu, caudate-putamen. mRNA expression was normalized to DOR-flox controls. *n* = 10–17 per genotype, circles for females and squares for males. Sex-grouped GFAP-DOR-KO and control DOR-flox mice were compared by Mann-Whitney analysis. *P*-values for the difference between genotypes are shown when significant (*p* < 0.05). **(C)** Gfap expression was 14-fold higher than Tubb3 expression in the purified astrocytes. **(D)** Gfap expression was 30-fold lower than Tubb3 expression in the effluent from the ACSA-2 beads. **(E)** Oprd1 expression was significantly reduced in purified astrocytes from GFAP-DOR-KO mice as compared to DOR-flox mice. Mann-Whitney test, *P*-value indicates a genotype difference. **(F)** Oprd1 expression is comparable in ACSA-2 beads effluents from GFAP-DOR-KO and DOR-flox mice. Mann-Whitney test. Means ± SEM are shown. See also Supplementary Table 1 for statistics.

The analysis of genomic DNA in GFAP-DOR-KO mice showed *Oprd1-flox* gene inactivation in the brain cortex, periaqueductal gray, and brainstem (Figure 1A right part). Gene inactivation was also evaluated by comparing *Oprd1* mRNA levels in sex-grouped GFAP-DOR-KO and DOR-flox control animals. Delta receptor transcript levels were reduced in the brain cortex, hippocampus, periaqueductal gray, and brainstem of GFAP-DOR-KO mice (Figure 1B, Supplementary Table 1). Two-way ANOVA analyses for the effects of genotype and sex were performed on each region. In the dorsal root ganglia, spinal cord, olfactory bulb, and Caudate putamen, there was no effect of genotype or sex. The cortex, hippocampus, PAG, and brainstem showed a genotype effect with a sex effect in brainstem (Supplementary Table 1).

Gene inactivation in purified astrocytes was determined by genomic DNA analysis of astrocytes isolated from the brain by anti-ACSA-2 magnetic beads separation. The floxed gene was deleted in astrocytes (Eluate) as compared to non-astrocyte (Effluent) cells from GFAP-DOR-KO mice (Figure 1A left part). *Oprd1* expression was analyzed in the isolated astrocytes. Astrocyte enrichment was evaluated by *Gfap* and *Tubb3* expression levels as markers for astrocytes and neurons, respectively. In the ACSA-2 magnetic bead eluates, Gfap vs. Tubb3 fold expression was 13.8 ± 3.74 (Figure 1C) while it was 0.034 ± 0.008 in magnetic bead effluents (Figure 1D), indicating a 400-fold enrichment for astrocytes in ACSA-2 beads eluates vs. effluents. *Oprd1* expression level was reduced in enriched astrocytes from GFAP-DOR-KO mice as compared to DOR-flox counterparts (Figure 1E) while it was comparable in effluents from GFAP-DOR-KO and DOR-flox animals (Figure 1F, Supplementary Table 1).

### The Absence of Astrocytic DOR Does Not Alter Neuropathic Mechanical Allodynia

**Figure 2:**
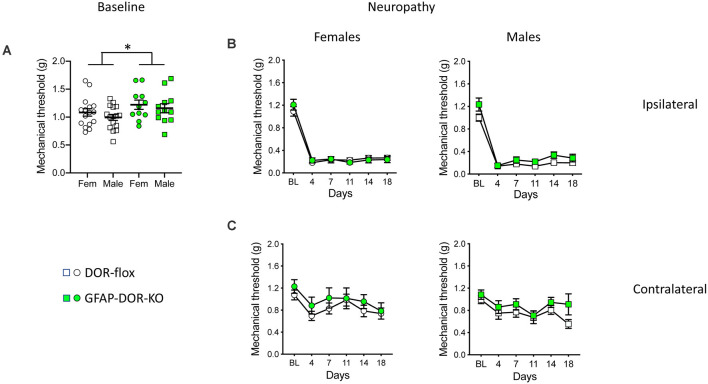
Mechanical neuropathic allodynia in GFAP-DOR-KO mice. **(A)** Mechanical sensitivity thresholds in GFAP-DOR-KO and DOR-flox mice. GFAP-DOR-KO mice showed overall higher nociceptive mechanical thresholds. There was no genotype difference when evaluated in separated sexes (Two-way ANOVA followed by Sidak’s multiple comparison tests). *, *p* < 0.05 GFAP-DOR-KO vs. DOR-flox. **(B)** Partial sciatic nerve ligation (pSNL) induced comparable mechanical allodynia at the ipsilateral side in GFAP-DOR-KO and DOR-flox mice of both sexes. **(C)** pSNL induced a mild allodynia at the contralateral side in both GFAP-DOR-KO and DOR-flox mice in the two sexes. *n* = 16 DOR-flox females, *n* = 11 GFAP-DOR-KO females, *n* = 16 DOR-flox males, *n* = 12 GFAP-DOR-KO males. Two-way repeated measures ANOVA followed by Sidak’s multiple comparison test. Data are expressed as mean ± SEM. See also Supplementary Table 2 for statistics.

Prior to inducing neuropathy, GFAP-DOR-KO mice showed overall elevated nociceptive mechanical thresholds as compared to their control DOR-flox littermates. However, this did not reach significance when evaluated in separated sexes (Figure 2A, Supplementary Table 2). We then examined the contribution of astrocytic DOR in neuropathic allodynia by using the pSNL model previously used to analyze other DOR mutant mouse lines (Nadal et al., 2006; Gaveriaux-Ruff et al., 2011; Nozaki et al., 2012; Martínez-Navarro et al., 2020). Mice with pSNL developed strong mechanical hypersensitivity at the ipsilateral side in both sexes and genotypes (Figure 2B, Supplementary Table 2). The three-way ANOVA analysis of mechanical sensitivity at the operated side indicated an effect of time but not sex or genotype for mechanical allodynia (Supplementary Table 2). When each sex was examined separately, pSNL led to a comparable allodynia in females of both genotypes. In males, there was an overall genotype effect that may be due to a higher baseline threshold, and there was no genotype difference at individual time points (Figure 2B, Supplementary Table 2). At the contralateral side, pSNL induced some allodynia which was less severe than at the ipsilateral side (Figure 2C, Supplementary Table 2). The three-way ANOVA indicated an effect of time and genotype, but no genotype difference was found when the genotype effect was evaluated in each sex separately (Supplementary Table 2). Overall, the results show no influence of astrocytic DOR deletion on neuropathic mechanical allodynia.

### Tolerance to SNC80-Induced Analgesia Is Abolished in Female DOR-GFAP-KO Mice Under Neuropathic Condition

**Figure 3:**
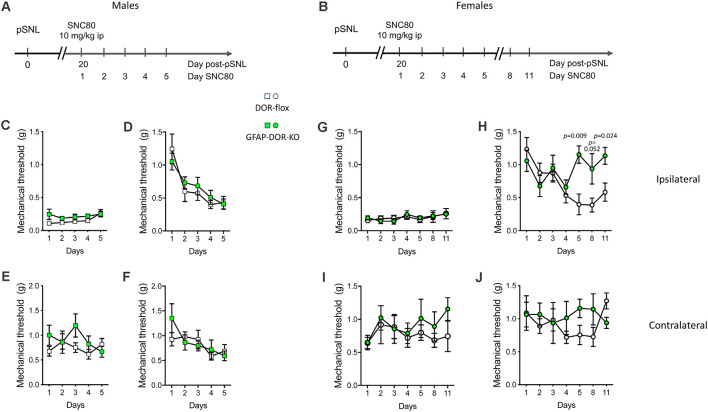
Analgesic tolerance to SNC80 is abolished in GFAP-DOR-KO female mice. **(A,B)** Experimental design in males and females. SNC80-induced analgesia and analgesic tolerance was scored in neuropathic GFAP-DOR-KO and DOR-flox males **(A)** and females **(B)**. **(C)** Mechanical sensitivity in males each day before SCN80 administration, ipsilateral side. **(D)** Mechanical sensitivity at ipsilateral side in males following SCN80 administration shows analgesic tolerance in each genotype and no genotype difference. **(E)** Sensitivity in males each day before SCN80 administration, contralateral side.** (F)** Sensitivity in males each day after SCN80 administration, contralateral side. *n* = 13 DOR-flox males, *n* = 11 GFAP-DOR-KO males, two-way repeated measures ANOVA and one-way repeated measures ANOVA on each genotype followed by Sidak’s multiple comparison tests. **(G)** Mechanical sensitivity at ipsilateral side in females each day before SCN80 administration. **(H)** Mechanical sensitivity following SCN80 administration shows that analgesic tolerance occurred in DOR-flox females but did not develop in GFAP-DOR-KO females. **(I)** Sensitivity in females each day before SCN80 administration, contralateral side.** (J)** Sensitivity in females each day after SCN80 administration, contralateral side. *n* = 10 DOR-flox females, *n* = 7 GFAP-DOR-KO females, mixed effect analysis for genotype comparison followed by Sidak’s multiple comparison test and mixed effect analysis of each genotype followed by Wilcoxon test. *P*-values for the difference between genotypes are shown when significant (*p* < 0.05). Data are expressed as mean ± SEM. See also Supplementary Table 3 for statistics.

We then examined whether astrocytic DOR did contribute to analgesia and analgesic tolerance elicited by the prototypal DOR agonist SNC80. It is known that DOR activation leads to weak antinociception in naïve animals but induces robust analgesia in chronic pain models (Berthiaume et al., 2020). Therefore, we scored SNC80-induced analgesia in neuropathic GFAP-DOR-KO and DOR-flox mice (see Figures 3A,B for experimental design). Mechanical sensitivity was determined daily for 5 days (males) or on days 1–5, 8 and 11 (females) before (Figures 3C,E,G,I) and following (Figures 3D,F,H,J) SCN80 injection. In males, SCN80 reversed mechanical allodynia after the first administration, and this analgesia gradually decreased over the 5 days of SNC80 treatment, demonstrating analgesic tolerance that was comparable in the two genotypes (Figure 3D, Supplementary Table 3). Mechanical sensitivity prior to SNC80 administration did not lower over days, indicating that analgesic tolerance occurred in the absence of hyperalgesia (Figure 3C, Supplementary Table 3). On the contralateral side, repeated SNC80 treatment did not lower mechanical sensitivity prior to its administration on days 2–5 (Figure 3E, Supplementary Table 3). This treatment also caused an overall decrease in sensitivity following its administration on days 2–5, but that did not reach significance in individual genotypes (Figure 3F, Supplementary Table 3).

In females, daily SNC80 doses did not elicit any opioid-induced hyperalgesia prior to SNC80 in any genotype under these experimental conditions, similarly to the males (Figure 3G, Supplementary Table 3). In DOR-flox females, SNC80 produced the expected anti-allodynic effect on day 1 of treatment, and analgesic tolerance developed from day 4 and remained until day 8. However, the GFAP-DOR-KO females showed no analgesic tolerance, and this absence of tolerance persisted until day 11 (Figure 3H, Supplementary Table 3). On the contralateral side, chronic SNC80 treatment produced no decrease in mechanical sensitivity when scored either before or after its administration (Figures 3I,J, Supplementary Table 3). Overall, this suggests that astrocytic DOR contributes to tolerance to delta opioid analgesia in females.

### Neuropathic Cold Allodynia Is Attenuated in DOR-GFAP-KO Mice

**Figure 4:**
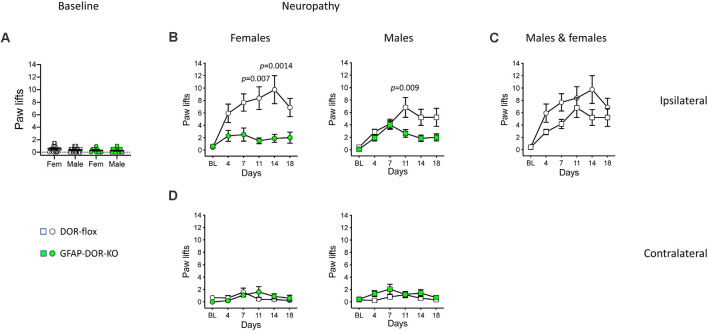
Cold neuropathic allodynia was attenuated in GFAP-DOR-KO mice. **(A)** Baseline paw responses on the cold plate in GFAP-DOR-KO and DOR-flox mice. GFAP-DOR-KO mice reacted comparably to DOR-flox control mice (Two-way ANOVA). **(B)** Paw responses at the ipsilateral side on the cold plate following pSNL. Cold allodynia was abolished in DOR-GFAP-KO females, with genotype differences on individual days 11 and 14. Cold allodynia was reduced in GFAP-DOR-KO males with a genotype difference on day 11 time-point. **(C)** The comparison of cold allodynia in DOR-flox females and males indicated no sex difference. **(D)** There was a modest cold allodynia at the contralateral side in males but not females, which did not reach significance at any individual time points. *n* = 16 DOR-flox females, *n* = 10 GFAP-DOR-KO females, *n* = 17 DOR-flox males, *n* = 14 GFAP-DOR-KO males. Two-way repeated measures ANOVA followed by Sidak’s multiple comparison test. Data are expressed as mean ± SEM. See also Supplementary Table 4 for statistics.

Before pSNL, DOR-GFAP-KO and DOR-flox mice showed comparable baseline responses on the cold plate (Figure 4A, Supplementary Table 4). Neuropathic cold allodynia was then analyzed for time, genotype, and sex effects. For the ipsilateral side, three-way ANOVA indicated strong effects of time and genotype (Supplementary Table 4). In DOR-flox females, pSNL elicited an important ipsilateral cold allodynia, that was abolished in DOR-GFAP-KO females (Figure 4B, Supplementary Table 4). In DOR-flox males, pSNL also elicited ipsilateral cold hypersensitivity (Figure 4B, Supplementary Table 4). The GFAP-DOR-KO males showed an attenuation of cold allodynia over time as compared to DOR-flox littermates (Figure 4B, Supplementary Table 4). The comparison of cold allodynia in DOR-flox females and males revealed no sex effect (Figure 4C, Supplementary Table 4). The three-way analysis indicated a modest time effect on cold response at the contralateral side (Figure 4D, Supplementary Table 4). Nerve lesion elicited no contralateral cold allodynia in females and a modest allodynia in males with a slight genotype difference that did not reach significance on individual day points (Figure 4D, Supplementary Table 4). Overall, cold allodynia was abolished in the cKO females and reduced in cKO males, revealing a key role of astrocytic DOR in neuropathic cold allodynia.

### Deletion of DOR in the Whole Body Aggravates Neuropathy-Induced Cold Allodynia in Females

**Figure 5:**
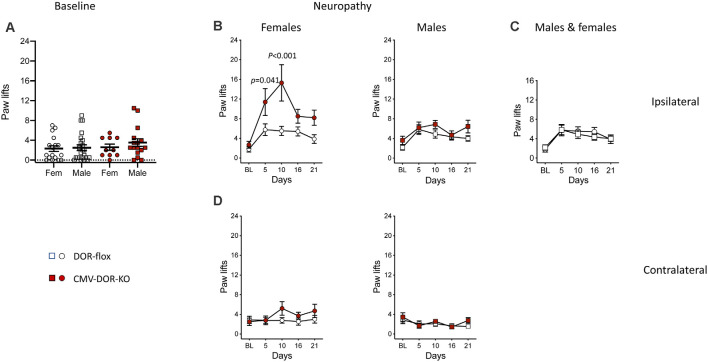
Cold neuropathic allodynia was aggravated in CMV-DOR-KO female mice. **(A)** Baseline paw responses on the cold plate were similar in CMV-DOR-KO and control DOR-flox mice (Two-way ANOVA). **(B)** Paw responses at the ipsilateral side. In females, cold allodynia was augmented in CMV-DOR-KO mice as compared to DOR-flox mice, and genotype difference was significant on individual days 5 and 10. In males, allodynia was comparable in the two genotypes. **(C)** Cold allodynia in DOR-flox females and males showed no sex effect. **(D)** There was cold allodynia at the contralateral sides in females and males of both genotypes. *n* = 17 DOR-flox females, *n* = 10 CMV-DOR-KO females, *n* = 23 DOR-flox males and *n* = 16 CMV-DOR-KO males. Two-way repeated measures ANOVA followed by Sidak’s multiple comparison test. *P*-values for the difference between genotypes are shown when significant (*p* < 0.05). Data are expressed as mean ± SEM. See also Supplementary Table 5 for statistics.

The above results indicate a major role for astrocytic DOR in the development of neuropathic cold allodynia. Previously, the role of DOR in neuropathic cold allodynia has been investigated by using conventional DOR KO mice, showing that DOR KO aggravated cold allodynia but the sex influence was not fully investigated (Nadal et al., 2006). Recently the influence of global DOR activity in neuropathy-induced heat hyperalgesia and mechanical allodynia was investigated using CMV-DOR-KO mice generated from crossing DOR-flox mice with CMV-Cre mice but cold allodynia was not reported (Martínez-Navarro et al., 2020). In this study, in view of the results on GFAP-DOR-KO animals, we examined whether DOR deletion in the whole body would exert a sex-specific effect on cold allodynia. We analyzed CMV-DOR-KO mice in the same pSNL model as for GFAP-DOR-KO animals. First, we determined whether whole body DOR deletion alters basal cold nociception. In basal condition, CMV-DOR-KO and DOR-flox control mice showed comparable reactions on the cold plate, with no sex effect (Figure 5A, Supplementary Table 5). Cold responses in neuropathic mice were scored at both sides and analyzed for time, genotype, and sex effect by three-way ANOVA. At the ipsilateral side, cold allodynia was observed in both sexes, and effects of the three factors were found (Supplementary Table 5). In females, cold allodynia was aggravated in CMV-DOR-KO mice as compared to DOR-flox mice (Figure 5B, Supplementary Table 5). In males, ipsilateral allodynia was comparable across genotypes (Figure 5B, Supplementary Table 5). The comparison of cold allodynia in DOR-flox female and male control mice revealed no sex effect (Figure 5C, Supplementary Table 5). There was no contralateral cold allodynia in females and males (Figure 5D, Supplementary Table 5). Globally these results suggest that DOR activation is protective against neuropathic cold allodynia in female mice.

## Discussion

We established a mouse conditional KO line for DOR in astrocytes. Using these mice, we tested whether the astrocytic DOR contributes to neuropathic allodynia as well as analgesia and analgesic tolerance. We showed that analgesia was not altered in the mutants, while analgesic tolerance was abolished in female mutants. In addition, astrocytic DOR does not participate in mechanical allodynia but contributes to cold allodynia while global DOR activity protects from cold allodynia.

### Role of Astrocytic DOR in SNC80 Analgesia and Analgesic Tolerance

We have found that SNC80 induces comparable analgesia in neuropathic GFAP-DOR-KO and DOR-flox control mice. This suggests that astrocytic DOR does not contribute to DOR analgesia. Together with the previous demonstration that DOR-mediated analgesia was independent of microglial activation in a rat neuropathic pain model (Mika et al., 2014), this indicates that DOR on glial cells is not mainly involved in analgesia. Other DOR populations were shown to play a major role in DOR analgesia, in Nav1.8 peripheral neurons in inflammatory and neuropathic pain (Gaveriaux-Ruff et al., 2011; Nozaki et al., 2012) and in forebrain GABAergic neurons in migraine (Dripps et al., 2020). Therefore, the implication of astrocytic-DOR in the analgesic effect of novel DOR agonists including biased agonists may allow to better characterize these novel molecules (Conibear et al., 2020).

Our new finding is that analgesic tolerance to SNC80 is abolished in GFAP-DOR-KO females while it is maintained in males. This suggests that astrocytic DOR contributes to DOR-mediated tolerance in females. Recently, we have shown that MOR deletion in microglia delayed analgesic tolerance to morphine, indicating a role for microglial MOR in this morphine chronic effect (Reiss et al., 2020). Also, astrocytic MOR activity was shown to be involved in MOR-dependent long-term potentiation in the hippocampus and in morphine-induced conditioned place preference, although tolerance was not studied (Nam et al., 2019). To our knowledge, this is the first description of a role of an astrocytic opioid receptor in analgesic tolerance. The mechanisms by which astrocytic DOR favors analgesic tolerance still need to be explored. They may be identified by investigating the signaling pathways already described in other cell types, such as DOR phosphorylation, beta-arrestin recruitment, G proteins and Regulators of G protein Signaling, and more broadly the DOR interactome (Dripps et al., 2018; Degrandmaison et al., 2020; Jimenez-Vargas et al., 2020; Mann et al., 2020). Specific signaling pathways were shown activated by DOR activation including the PI3K pathway in human astrocytes cultures (Husain et al., 2017). DOR stimulation did also upregulate the astrocytic excitatory amino acid transporters (EAATs) in astrocyte cultures, suggesting decreased glutamate levels and neuroprotection (Liang et al., 2014). Repeated DOR triggering in astrocytes was shown to inhibit MMP-2 secretion and TNF-α production in cultures from optic nerve head astrocytes (Akhter et al., 2013; Zaidi et al., 2020) and in the sciatic nerve of neuropathic male rats (Vicario et al., 2016). However, whether these DOR-associated events or pathways are equally involved in male and female cells has not been clarified yet. Therefore, these previously described consequences of DOR activation may be explored in both sexes of the astrocytic DOR cKO animals.

### Astrocytic DOR Contributes to Neuropathic Cold Allodynia

The present study shows a slight increase in baseline mechanical threshold in GFAP-DOR-KO mice. In a previous study, CMV-DOR-KO and Nav1.8-DOR-KO male animals had a small decrease in the mechanical threshold. Altogether, this suggests that DOR activity in the whole body and in nociceptive neurons produces moderate endogenous analgesia on mechanical nociception while astrocytic DOR would rather increase mechanical sensitivity.

This work shows no change in basal cold sensitivity in the two DOR mutant mouse lines. There was also no alteration of basal cold reactions in Nav1.8-DOR-KO mice. Globally, this indicates that DOR endogenous activity does not mainly influence sensitivity to cold in non-neuropathic conditions.

We have found that astrocytic DOR deleted mice have an attenuated neuropathic cold allodynia and a similar mechanical allodynia as compared to DOR-flox mice, indicating that DOR in astrocytes worsens cold allodynia selectively. The role of endogenous DOR activity in neuropathic mechanical and cold pain was investigated previously using conventional DOR KO animals and the same pSNL paradigm as used in this study. These DOR KO mice had an aggravated hypersensitivity as compared to wild-type mice, which revealed that DOR activity was protective against neuropathic pain (Nadal et al., 2006). More recently, the role of a DOR global tone in neuropathy-induced mechanical allodynia and heat hyperalgesia was demonstrated by using CMV-DOR-KO mice but cold allodynia was not been reported (Martínez-Navarro et al., 2020). Altogether, the past and present results demonstrate an exacerbated cold allodynia in mice without DOR and hence that global DOR endogenous activity protects from neuropathic cold pain, especially in females. Specific DOR populations were shown to control pain in preclinical models. DOR in peripheral Nav1.8-positive nociceptive neurons and in immune cells were reported to protect against neuropathic pain (Gaveriaux-Ruff et al., 2011; Nozaki et al., 2012; Celik et al., 2016; Machelska and Celik, 2020). Also, DOR on forebrain GABAergic neurons were protective from migraine in the nitroglycerin model (Dripps et al., 2020). Peripheral DOR activation was more effective on neuropathic mechanical allodynia than heat hypersensitivity (Gaveriaux-Ruff et al., 2011; Labuz et al., 2016). The impact of peripheral DOR on cold allodynia has been evaluated in Nav1.8-DOR-KO mice and showed that endogenous DOR activity at these neurons protects from neuropathic cold allodynia (Gaveriaux-Ruff et al., 2011). Therefore, the present results showing that astrocytic DOR deletion attenuates neuropathic cold allodynia is the first demonstration of a DOR population that worsens cold pain. This suggests that the endogenous activation of astrocytic DOR would activate pathways that lead to cold allodynia. The mechanisms involved in this promotion of cold allodynia remain to be identified (MacDonald et al., 2020).

### The GFAP-DOR-KO Mouse Model

In GFAP-DOR-KO mice, we showed *Oprd1* gene deletion and decreased *DOR* expression in purified astrocytes from but not non-astrocytic cells present in the effluent from ACSA-2 beads. Gfap-Cre mouse lines have been shown efficient tools for gene inactivation in astrocytes and the Mouse Genetic Institute lists 27 different Gfap driver mouse lines. However, some debate has been raised on their cell specificity, in particular regarding small neuron populations (Guttenplan and Liddelow, 2019; Yu et al., 2020). Among the Gfap-Cre lines, the Tg(GFAP-cre)8Gtm line used in the present study was frequently employed and has been referenced in more than 40 publications, a part of which demonstrated the astrocytes specificity, see Chhatbar et al. (2018) for example. Other astrocyte deleter lines were used to inactivate genes in astrocytes, including the tamoxifen-inducible Cx30-ERT2 line (Tertil et al., 2021). However, tamoxifen was shown to induce cellular stress in the nervous system. Therefore, we used a strategy that did not require tamoxifen administration. Also, astrocytes show a great diversity in terms of morphology, density in the different brain regions and activation patterns, and a degree of complexity lies in the fact that the role of astrocytic DOR may vary in the multiple regions of the nervous system (Khakh and Deneen, 2019; Sofroniew, 2020). Therefore, in the future, the use of mouse models targeting astrocytic DOR in specific brain regions, by using novel mouse lines or viral approaches, may further contribute to the understanding of astrocytic DOR implication in response to pain and opiates.

### Sex Difference for the Role of Astrocytic DOR

Our results show exacerbated neuropathic cold allodynia in DOR full KO females, attenuated cold allodynia in both GFAP-DOR-KO females and males as well as the abolishment of analgesic tolerance in GFAP-DOR-KO females. The effect of sex on pain is clinically known and is now better considered in preclinical models (Mogil, 2012; Shansky and Murphy, 2021).

Studies in rodents have reported either sex difference or no sex difference in pain behaviors and analgesia, a part of which may be strain-dependent (Mogil, 2020). The mechanisms leading to sex difference in neuropathic pain encompass differential nociceptive neuron transcriptome (Mecklenburg et al., 2020), translatome (Tavares-Ferreira et al., 2020), and preferential involvement of macrophages/microglia in males and T lymphocytes in females, although not all studies concur to this conclusion (see Price and Ray, 2019; Mogil, 2020; Gregus et al., 2021 for recent reviews). Neutrophils have also been reported to account for sex differences in pain and opioid-mediated analgesia (Scheff et al., 2018, 2019). Regarding specific genes, the prolactin system was found to contribute to pain in females but not males (Chen et al., 2020). Also, differences in neuroinflammation in the central nervous system were shown involved in opioid analgesia and tolerance (Doyle et al., 2017; Eidson and Murphy, 2019).

Considering opioid receptor KO mice, opioid-mediated mild stress-induced analgesia was lowered in constitutive DOR KO and MOR KO females but not males (Contet et al., 2006) while opioid-induced hyperalgesia was comparable in female and male wild-type mice and abolished in MOR KO animals of both sexes (Roeckel et al., 2017). The CMV-DOR-KO mice had aggravated neuropathic mechanical allodynia as compared to DOR-flox mice in both sexes (Martínez-Navarro et al., 2020). Overall, the analysis of DOR expression in GFAP-DOR-KO did not show a main effect of sex in the regions analyzed. The attenuated cold allodynia and analgesic tolerance in GFAP-DOR-KO females may be due to lower activation of astrocytes by neuropathy as previously described in rats (Gutierrez et al., 2013) and mice (Vacca et al., 2014, 2021). More broadly, the molecular signature underlying sex difference in neuropathic cold pain and analgesic tolerance in GFAP-DOR-KO mice may be further explored by omics approaches.

### Human vs. Mouse Models

The pioneering work by Marco Loggia and colleagues has revealed glial activation in the brain of chronic pain patients by using imaging technology (Loggia et al., 2015; Jung et al., 2020). On the other hand, preclinical animal models have enabled us to advance in understanding the role of astrocytes in pain and opioid actions in homogenous conditions of age, environment, and treatment. Preclinical research provides a number of tools that allow to conditionally delete or express specific genes in astrocytes, visualize or manipulate astrocytes, profile gene expression or molecular networks, or assess behaviors (Yu et al., 2020). However, significant differences were reported between human and mouse astrocytes, including larger size, increased morphological complexity as well as differing subtypes defined by their expression profiles amongst other techniques (Oberheim et al., 2009; Ji et al., 2019; Escartin et al., 2021). One way to address the role of DOR in human astrocytes would be the use of human astrocytes derived from induced pluripotent stem cells (iPSC) *in vitro*, although *in vitro* culture induces astrocytes that differ from *in vivo* astrocytes (Barbar et al., 2020; Franklin et al., 2021; Peteri et al., 2021). Of note, human iPSC-derived astrocytes could be grafted into the mouse brain and retain human-specific features (Preman et al., 2020). As sex differences were found in this study, these novel approaches may be used on human astrocytes derived from individuals of both sexes.

### Perspectives

The first results on astrocytic DOR involvement in neuropathic pain and opioid analgesic tolerance reported here open the way to the exploration of the involvement of this DOR population in other pain paradigms including migraine (Dripps et al., 2020). Also, the role of astrocytic DOR in descending pain circuits may be explored (Price and Ray, 2019), as well as its impact on affective pain and on the anxio-depressive consequences of neuropathic pain (Birdsong et al., 2019; Cahill et al., 2020; Wahis et al., 2021). In addition, interactions between DOR and MOR systems have been described. Notably, glial activation and augmented cell surface DOR participated in enhancing deltorphin-induced analgesia following prolonged morphine administration (Holdridge et al., 2007). Furthermore, simultaneous activation of MOR and DOR was shown to reduce both allodynia and astrocytic gap junction protein connexin 43 in a neuropathic rat model (Vicario et al., 2019). Therefore, the impact of astrocytic-DOR on morphine analgesic tolerance and hyperalgesia (Cahill and Taylor, 2017) may be explored using the astrocytic DOR KO mouse line. As sex differences were found here for DOR-mediated chronic pain processing, the above questions are expected to be explored by considering both sexes. More broadly, the role of astrocytic-DOR in CNS inflammation in chronic pain condition (Giovannoni and Quintana, 2020), in astrocytes-neurons and astrocytes-microglia interactions (Greenhalgh et al., 2020; Vainchtein and Molofsky, 2020), and in astrocytes communication with immune cells from the periphery (Gregus et al., 2021) could allow to better understand the relationships between astrocytes and other cells involved in opioid effects and chronic pain.

## Data Availability Statement

The original contributions presented in the study are included in the article/Supplementary Material, further inquiries can be directed to the corresponding author.

## Ethics Statement

The animal study was reviewed and approved by Com’Eth, Comité d’Ethique pour l’Expérimentation Animale IGBMC-ICS, licence N° 17 and by Comité Etico de Experimentacion Animal del PRBB (CEEA-PRBB) with agreement number DAM-4917.

## Author Contributions

DR, HM, EA, MM-N, YX, RM, DC, and CG-R designed experiments, analyzed data, and contributed to the writing of the manuscript. DR, HM, EA, MM-N, DC, and YX performed experiments. YH contributed to the writing of the manuscript. All authors contributed to the article and approved the submitted version.

## Conflict of Interest

The authors declare that the research was conducted in the absence of any commercial or financial relationships that could be construed as a potential conflict of interest.

## Publisher’s Note

All claims expressed in this article are solely those of the authors and do not necessarily represent those of their affiliated organizations, or those of the publisher, the editors and the reviewers. Any product that may be evaluated in this article, or claim that may be made by its manufacturer, is not guaranteed or endorsed by the publisher.
