# Supplementary material for: Delta Opioid Receptor in Astrocytes Contributes to Neuropathic Cold Pain and Analgesic Tolerance in Female Mice
Source: Front Cell Neurosci. 2021 Sep 16;15:745178. doi: 10.3389/fncel.2021.745178 (PMC8483180; doi:10.3389/fncel.2021.745178)
Supplement: Supplementary file 1 [file Data_Sheet_1.PDF]

Table S1. Statistical analyses for data in figure 1

Figure panel	Assay	Statistical test, findings
Figure 1B	RT-PCR	Two-way ANOVA
	DRG*	Genotype $p = 0.802$
		Sex $p = 0.250$
		Interaction $p = 0.474$
	Spinal cord	Genotype $p = 0.952$
		Sex $p = 0.648$
		Interaction $p = 0.934$
	Olfactory bulb	Genotype $p = 0.168$
		Sex $p = 0.580$
		Interaction $p = 0.234$
	Cortex	Genotype $p = 0.001$
		Sex $p = 0.125$
		Interaction $p = 0.440$
	Caudate putamen	Genotype $p = 0.752$
		Sex $p = 0.076$
		Interaction $p = 0.876$
	Hippocampus	Genotype: $p = 0.008$
		Sex: $p = 0.942$
		Interaction $p = 0.895$
	PAG*	Genotype: $p = 0.022$
		Sex: $p = 0.358$
		Interaction $p = 0.726$
	Brainstem	Genotype: $p = 0.041$
		Sex: $p = 0.049$
		Interaction $p = 0.594$
Figure 1C	RT-PCR astrocytes	Mann-Whitney test
	Fold Gfap vs Tubb3	$p = 0.847$
Figure 1D	RT-PCR effluents	Mann-Whitney test
	Fold Gfap vs Tubb3	$p = 0.857$
Figure 1E	RT-PCR astrocytes	Mann-Whitney test
		Genotype: $p = 0.029$
Figure 1F	RT-PCR effluents	Mann-Whitney test
		Genotype: $p = 0.876$

* DRG, dorsal root ganglia; PAG, periaqueductal gray.

Table S2. Statistical analysis for mechanical sensitivity in GFAP-DOR-KO and DOR-flox mice

Figure panel	Assay	Statistical test, findings
Figure 2A	Baseline	Two-way ANOVA<br>Genotype: $p = 0.035$<br>Sex: $p = 0.284$
Figure 2B	pSNL*<br>Ipsilateral side	Three-way repeated-repeated-measures ANOVA<br>Time: $p < 0.001$<br>Genotype: $p = 0.056$<br>Sex: $p = 0.192$
Figure 2B	pSNL<br>Ipsilateral side<br>Females	Two-way repeated-measures ANOVA<br>Time: $p < 0.001$<br>Genotype: $p = 0.806$
Figure 2B	pSNL<br>Ipsilateral side<br>Males	Two-way repeated-measures ANOVA<br>Time: $p < 0.001$<br>Genotype: $p = 0.009$
Figure 2C	pSNL<br>Contralateral side	Three-way repeated-measures ANOVA<br>Time: $p < 0.001$<br>Genotype: $p = 0.037$<br>Sex: $p = 0.185$
Figure 2C	pSNL<br>Contralateral side<br>Females	Two-way repeated-measures ANOVA<br>Time: $p = 0.017$<br>Genotype: $p = 0.180$
Figure 2C	pSNL<br>Contralateral side<br>Males	Two-way repeated-measures ANOVA<br>Time: $p = 0.008$<br>Genotype: = 0.110

*pSNL, partial sciatic nerve ligation

Table S3. Statistical analysis of SCN80 analgesia and analgesic tolerance in GFAP-DOR-KO and DOR-flox mice

Figure panel	Assay	Statistical test, findings
Figure 3C	Males Ipsilateral<br>Mechanical sensitivity<br>before SNC80	Two-way repeated-measures ANOVA<br>Time: $p = 0.041$<br>Genotype: $p = 0.077$
Figure 3D	Males Ipsilateral<br>Mechanical sensitivity<br>after SNC80	Two-way repeated-measures ANOVA<br>Time: $p < 0.001$<br>Genotype: $p = 0.808$
	DOR-flox males<br>Tolerance to analgesia	One-way repeated-measures ANOVA<br>Time: $p = 0.0015$
	GFAP-DOR-KO males<br>Tolerance to analgesia	One-way repeated-measures ANOVA<br>Time: $p = 0.005$
Figure 3E	Males Contralateral<br>Mechanical sensitivity<br>before SNC80	Two-way repeated-measures ANOVA<br>Time: $p = 0.273$<br>Genotype: $p = 0.278$
Figure 3F	Males Contralateral<br>Mechanical sensitivity<br>after SNC80	Two-way repeated-measures ANOVA<br>Time: $p = 0.014$<br>Genotype: $p = 0.707$
	DOR-flox males<br>Mechanical sensitivity	One-way repeated-measures ANOVA<br>Time: $p = 0.117$
	GFAP-DOR-KO males<br>Mechanical sensitivity	One-way repeated-measures ANOVA<br>Time: $p = 0.097$
Figure 3G	Females Ipsilateral<br>Mechanical sensitivity<br>before SNC80	Mixed-effects model<br>Time: $p = 0.505$<br>Genotype: $p = 0.866$
Figure 3H	Females Ipsilateral<br>Mechanical sensitivity<br>after SNC80	Mixed-effects model<br>Time: $p = 0.032$<br>Genotype: $p = 0.055$
	DOR-flox females<br>Tolerance to analgesia	Mixed-effects model<br>Time: $p = 0.017$
	GFAP-DOR-KO females<br>Tolerance to analgesia	Mixed-effects model<br>Time: $p = 0.232$
Figure 3I	Females Contralateral<br>Mechanical sensitivity<br>before SNC80	Mixed-effects model<br>Time: $p = 0.415$<br>Genotype: $p = 0.355$

Table S3. Statistical analysis of SCN80 analgesia and analgesic tolerance in GFAP-DOR-KO and DOR-flox mice (continued)

Figure panel	Assay	Statistical test, findings
Figure 3J	Females Contralateral<br>Mechanical sensitivity<br>after SNC80	Mixed-effects model<br>Time: $p = 0.815$<br>Genotype: $p = 0.334$
	DOR-flox females<br>Mechanical sensitivity	Mixed-effects model<br>Time: $p = 0.300$
	GFAP-DOR-KO females<br>Mechanical sensitivity	Mixed-effects model<br>Time: $p = 0.931$

Table S4. Statistical analysis for cold sensitivity in GFAP-DOR-KO and DOR-flox mice

Figure panel	Assay	Statistical test, findings
Figure 4A	Baseline	Two-way ANOVA<br>Genotype: $p = 0.077$<br>Sex: $p = 0.298$
Figure 4B	pSNL*<br>Ipsilateral side	Three-way repeated-measures ANOVA<br>Time: $p < 0.001$<br>Genotype: $p < 0.001$<br>Sex $p = 0.192$
Figure 4B	pSNL<br>Ipsilateral side<br>Females	Two-way repeated-measures ANOVA<br>Time: $p = 0.006$<br>Genotype: $p = 0.0026$
Figure 4B	pSNL<br>Ipsilateral side<br>Males	Two-way repeated-measures ANOVA<br>Time: $p < 0.001$<br>Genotype: $p = 0.025$
Figure 4C	pSNL<br>Ipsilateral side<br>Males vs females	Two-way repeated-measures ANOVA<br>Time: $p < 0.001$<br>Sex $p = 0.0697$
Figure 4D	pSNL<br>Contralateral side	Three-way repeated-measures ANOVA<br>Time: $p = 0.002$<br>Genotype: $p = 0.112$<br>Sex: $p = 0.353$
Figure 4D	pSNL<br>Contralateral side<br>Females	Two-way repeated-measures ANOVA<br>Time: $p = 0.087$<br>Genotype: $p = 0.805$
Figure 4D	pSNL<br>Contralateral side<br>Males	Two-way repeated-measures ANOVA<br>Time: $p = 0.044$<br>Genotype: $p = 0.045$

*pSNL, partial sciatic nerve ligation

Table S5. Statistical analysis of cold response in CMV-DOR-KO and DOR-flox mice

Figure panel	Assay	Statistical test, findings
Figure 5A	Baseline	Two-way ANOVA<br>Genotype: $p = 0.346$<br>Sex: $p = 0.424$
Figure 5B	pSNL*<br>Ipsilateral side	Three-way repeated-measures ANOVA<br>Time: $p < 0.001$<br>Genotype: $p < 0.001$<br>Sex: $p = 0.0092$
Figure 5B	Females<br>pSNL<br>Ipsilateral side	Two-way repeated-measures ANOVA<br>Time: $p < 0.001$<br>Genotype: $p = 0.0013$
Figure 5B	Males<br>pSNL<br>Ipsilateral side	Two-way repeated-measures ANOVA<br>Time: $p < 0.001$<br>Genotype: $p = 0.104$
Figure 5C	pSNL<br>Ipsilateral side	Two-way repeated-measures ANOVA<br>Time: $p < 0.001$<br>Sex $p = 0.785$
Figure 5D	pSNL<br>Contralateral side	Three-way repeated-measures ANOVA<br>Time: $p = 0.107$<br>Genotype: $p = 0.177$<br>Sex: $p = 0.030$
Figure 5D	Females<br>pSNL<br>Contralateral side	Two-way repeated-measures ANOVA<br>Time: $p = 0.170$<br>Genotype: $p = 0.261$
Figure 5D	Males<br>pSNL<br>Contralateral side	Two-way repeated-measures ANOVA<br>Time: $p = 0.010$<br>Genotype: $p = 0.539$

*pSNL, partial sciatic nerve ligation
